# FROM PERSONAL TO GLOBAL: CULTIVATING YOUR GERONTOLOGICAL VOICE

**DOI:** 10.1093/geroni/igz038.3136

**Published:** 2019-11-08

**Authors:** Leah M Janssen

**Affiliations:** Miami University & Scripps Gerontology Center, Oxford, Ohio, United States

## Abstract

From intro-level undergraduates to advanced graduate students in gerontology, understanding and developing a “gerontological voice” is often an elusive concept and challenging process to articulate. How we view, understand, and give voice to aging-related issues is influenced by a number of different factors: Micro-level factors (e.g., personality, personal experiences with older adults, and personal interests and values); meso-level factors (e.g., coursework in aging and academic major/discipline); and, macro-level factors (e.g., societal and cultural values, media, activism, and advocacy). As an adaptable teaching exercise for all levels of students, this poster presents a novel framework to support students’ exploration and cultivation of their unique gerontological voice. Through this three-level scaffolded discovery, students across disciplines strengthen their awareness and understanding of their distinct voice and build confidence to activate around aging-related issues pertinent to their specific interests and passions. From personal to global, this guided introspective exercise provides an opportunity for students to focus and embed their voice into the larger tapestry of gerontology, an inclusive community rich with perspective and diversity. Encouraging the development of students is an essential part of building social capital which is instrumental to the creation of strong networks to serve older adults through a multitude of disciplines.

